# Genetic associations between voltage-gated calcium channels and autism spectrum disorder: a systematic review

**DOI:** 10.1186/s13041-020-00634-0

**Published:** 2020-06-22

**Authors:** Xiaoli Liao, Yamin Li

**Affiliations:** 1 Xiangya Nursing School, Central South University, Changsha, Hunan China; 2 Clinical Nursing Teaching and Research Section, The Second Xiangya Hospital, Central South University, Changsha, Hunan China

**Keywords:** Autism spectrum disorder, Voltage-gated calcium channels, Calcium signaling, Calcium pathway

## Abstract

**Objectives:**

The present review systematically summarized existing publications regarding the genetic associations between voltage-gated calcium channels (VGCCs) and autism spectrum disorder (ASD).

**Methods:**

A comprehensive literature search was conducted to gather pertinent studies in three online databases. Two authors independently screened the included records based on the selection criteria. Discrepancies in each step were settled through discussions.

**Results:**

From 1163 resulting searched articles, 28 were identified for inclusion. The most prominent among the VGCCs variants found in ASD were those falling within loci encoding the α subunits, CACNA1A, CACNA1B, CACNA1C, CACNA1D, CACNA1E, CACNA1F, CACNA1G, CACNA1H, and CACNA1I as well as those of their accessory subunits CACNB2, CACNA2D3, and CACNA2D4. Two signaling pathways, the IP3-Ca^2+^ pathway and the MAPK pathway, were identified as scaffolds that united genetic lesions into a consensus etiology of ASD.

**Conclusions:**

Evidence generated from this review supports the role of VGCC genetic variants in the pathogenesis of ASD, making it a promising therapeutic target. Future research should focus on the specific mechanism that connects VGCC genetic variants to the complex ASD phenotype.

## Background

### Autism spectrum disorder

Autism spectrum disorder (ASD) is a neurodevelopmental disorder characterized by deficits in social-communication skills along with repetitive stereotypic behaviors that manifest in early postnatal life [24]. The incidence of ASD has markedly increased 10 to 17% per year during the last two decades [4], affecting approximately 1 out of 68 children in the United States [3]. The rapid rise in the prevalence of ASD has stimulated numerous research interests into the potential etiology of this disorder, but the underlying molecular and cellular mechanisms have yet to be determined. Advances in human genetics and sequencing technologies have identified a stunning number of newly arising mutations associated with ASD [15], some of which are located in genes encoding voltage-gated calcium channels (VGCCs).

### Voltage-gated calcium channels

Voltage-gated calcium channels (VGCCs) are transmembrane proteins that activate in response to depolarization of the cell membrane and mediate the flux of calcium (Ca^2+^) into excitable cells [1, 20]. The VGCCs can be divided into high voltage-activated channels (HVA) and low voltage-activated channels (LVA) based on their electrophysiological properties [6]. HVA channels include L-, N-, P−/Q- and R-type channels, and LVA channels are also known as T-type calcium channels [13, 44, 55].

The L-type calcium channel is activated by a strong depolarization voltage and inactivated with a slow time course and is expressed in neurons, endocrine, cardiac, and smooth muscle [12]. The N-, P−/Q-, and R-type calcium channels also require strong depolarization for activation and are expressed mainly in neurons [28]. The T-type calcium channel is opened with weak depolarization voltages and remains active for a short time and is widely expressed in various cell types [49].

VGCCs are hetero-oligomeric proteins composed of a pore-forming α1 subunit and a group of auxiliary subunits known as α2δ and β [14, 16]. The α1 subunit is encoded by the CACNA1x genes (CACNA1A to CACNA1I and CACNA1S), and there are 10 isoforms in the human genome [7, 20]. The L-type channels, including Ca_v_1.1, Ca_v_1.2, Ca_v_1.3, and Ca_v_1.4, are encoded by CACNA1S, C, D, and F, respectively [8]. The P/Q-, N- and R-type channels, corresponding to Ca_v_2.1, Ca_v_2.2, and Ca_v_2.3, are encoded by CACNA1A, B and E, respectively [8]. The T-type channels, including Ca_v_3.1, Ca_v_3.2, and Ca_v_3.3, are encoded by CACNA1G, H and I, respectively [35]. Four genes exist for the Ca_v_α2δ subunits (CACNA2D1–4) and encode Ca_v_α2δ-1-4 proteins. Four genes exist for the Ca_v_β subunits (CACNB1–4) and encode Ca_v_β1–4 proteins.

Both the Ca_v_1 and Ca_v_2 channels form multiprotein complexes comprising the Ca_v_α1 pore-forming co-assembly with one of four α2δ subunits and one of four β subunits. The Ca_v_3 channels form functional channels using the α1 subunits alone but may also associate with other proteins. The kinetics, voltage-dependence, and pharmacological properties of the calcium channels are principally determined by the α1 subunits [20]. Although α1 subunits determine the main properties of the calcium channels, their functions are modified by the two auxiliary subunits. These auxiliary subunits have profound effects on the biophysical properties and membrane targeting of the α1 subunit [14, 16]. The different channel isoforms and possible combinations make for considerable potential diversity in the properties and function of the calcium channels.

### Objectives

Although the association between VGCCs and ASD has been reported in both correlation studies and informative reviews, no systematic review has been conducted to comprehensively synthesize the literature to determine whether this is fully supported by the totality of all available evidence. Therefore, the present review aimed to systematically summarize existing evidence regarding the role of VGCC genetic variants in the etiology of ASD.

## Methods

### Literature search

Electronic research was conducted to identify pertinent English articles examining the genetic associations between VGCCs and ASD. Three online databases, PubMed, Embase, and Web of Science, were searched using the following search terms: (ASD OR autism OR autistic disorder OR autism spectrum disorder OR Asperger syndrome OR pervasive developmental disorder) AND (Ca^2+^ channel OR Ca^2+^ signal OR Ca^2+^ pathway OR Ca^2+^ homeostasis OR calcium channel OR calcium signal OR calcium pathway OR calcium homeostasis OR CACNA1* OR CACNB* OR CACNA2D*).

A brief review of the articles was used to identify preliminary keywords. No restrictions were placed on the study location or the publication year. All authors achieved a consensus on the search strategies. References cited by all eligible citations and previous reviews were manually searched for additional relevant studies that might have been omitted.

### Study selection

The following criteria were required for articles to be eligible: focused on associations between VGCC gene variants and ASD; presented variants in the VGCC gene or reported deregulation of calcium signal; performed on human beings or human cells. Reviews, editorials and commentaries were excluded.

### Data extraction

Two authors independently extracted key information from each eligible study using a predefined abstraction form. Extracted data included first author, publication year, country setting, sample size, studied gene, identified mutation, adopted methods, and main findings.

### Data synthesis

Study characteristics were descriptively reported and study results were qualitatively reviewed due to the considerable heterogeneities across the included studies.

## Results

### Literature search

**Fig. 1:**
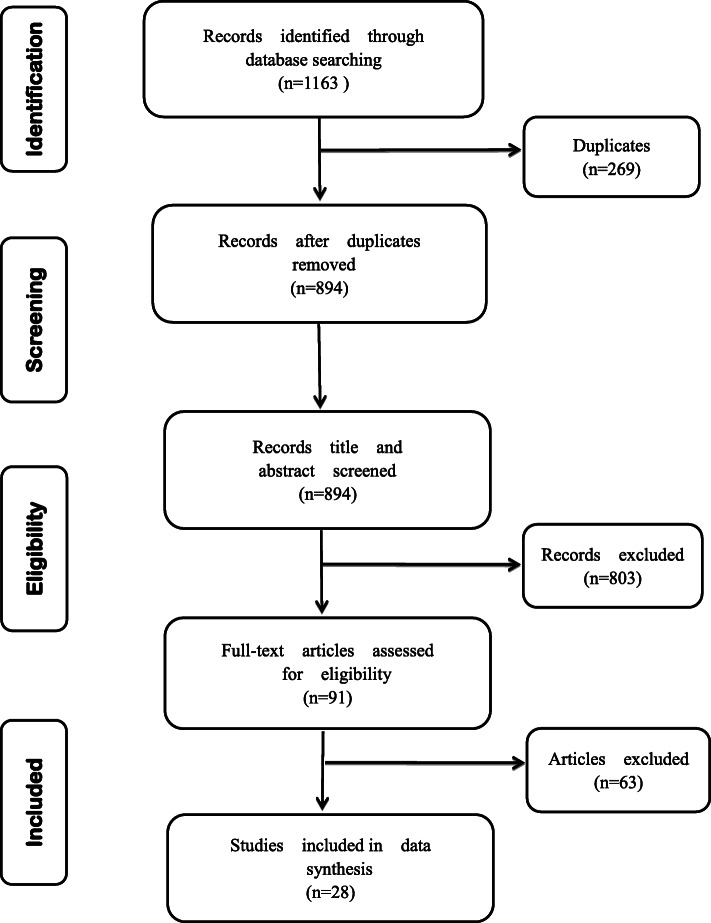
Study selection flowchart

Figure 1 presented the process of literature screening. A total of 1163 records were generated from the literature search. After eliminating 269 duplicates, 894 were considered as potentially relevant for subsequent analysis. The titles and abstracts of the remaining 894 were preliminary checked by two authors, and 803 were excluded as lacking of relevance. The full-text of the leaving 91 citations were further appraised independently by two authors, and 63 were excluded as failing to meet the selection criteria. A final total of 28 articles were selected as source documents for this review.

### Study characteristics

Table 1 summarized the key information of included studies. Animal models of psychiatric disorders are often unable to reflect typical features of human psychiatric disorders, although there is consensus on some animal correlates of clinical manifestations [2]. While there is an increasing number of ASD mouse models, studies regarding the role of VGCCs in the etiology of ASD are sparse [30]. Thus, the present review focused exclusively on human studies regarding the genetic association between VGCCs and ASD.

**Table 1:** Key information of included studies

Reference	Country	Sample	Gene	Mutation	Gain/loss of function	Method	Main Findings
Damaj et al. [10]	Canada	16 individuals with ASD-like behavioral deficits	CACNA1A	(1)deletion (2) point mutation	loss-of-function	whole genome sequencing	Results from sequencing revealed one CACNA1A gene deletion as well as two deleterious CACNA1A point mutations including one known stop-gain and one new frameshift variant and a new splice-site variant, which pointed to an association between CACNA1A variants and ASD symptoms.
Pinggera et al .[33]	Austria	tsA-201 cells	CACNA1D	de novo variant	gain-of-function	whole-cell patch-clamp	The study identified two de novo mutation, p.A749G and p.G407R, in ASD subjects, both demonstrated a pronounced gain-of-function.
Splawski et al .[43]	USA	461ASD 480controls	CACNA1H	rare missense variant	lose of function	(1) Genotypic Analyses (2) DNA Sequence Analyses	The study identified missense mutations in CACNA1H in 6 of 461 individuals with ASD.
Splawski et al .[42]	USA	13 TS 13 controls	CACNA1C	de novo missense variant	gain-of-function	(1) Genotypic Analyses (2) DNA Sequencing	The result showed that the CACNA1C gain-of-function mutation causes the diverse physiological and developmental defects in TS.
Li et al .[23]	China	553 trios	CACNA1A	SNPs		(1) SNP selection (2) SNP genotyping	The study identified association of rs7249246 and rs12609735 with ASD.
Strom et al .[45]	USA	284 trios	CACNA1G	SNPs		(1) SNP selection (2) SNP genotyping	Markers within an interval containing the gene, CACNA1G, were found to be associated with ASD at a locally significant level.
Smith et al .[41]	USA	(1)69 ASD (2)35 parents (3)89 CEU HapMap controls	CACNA2D4	CNVs		Microarray analyses	The study identified a rare homozygous deletion in a male proband that removed one copy of the CACNA2D4 calcium channel genes(12p13.33)
Pinggera et al .[34]	Germany	(1)1ASD (2)tsA-201 cells	CACNA1D	de novo missense variant	gain-of-function	(1) DNA Sequencing (2)whole-cell patch-clamp	The study identified a de novo missense mutation in CACNA1D (V401L) in a patient with ASD.
Breitenkamp et al .[5]	Germany	(1)155 ASD (2)259 controls	CACNB2	rare missense variant	gain-of-function	(1) Genotypic Analyses (2) DNA Sequencing	The study identified three missense mutations in CACNB2 gene in ASD subjects that result in a decelerated inactivation of the Cav1.2 subunit
Li et al .[23]	China	553 trios	CACNA1C	SNPs		(1) SNP selection (2) SNP genotyping	The result found a nominal significant association between two SNPs (rs1006737 and rs4765905) in CACNA1C and ASD.
Lu et al .[25]	USA	2781 trios	(1)CACNA1C (2)CACNA1G (3)CACNA1I	SNPs		(1) SNP selection (2) SNP genotyping	Four SNPs in three CCGs were associated with ASD. One, rs10848653, is located in CACNA1C.Two others, rs198538 and rs198545, located in CACN1G, and a fourth, rs5750860, located in CACNA1I.
Iossifov et al .[21]	USA	343 families with ASD	CACNA2D3	de novo variant		DNA Sequencing	The result identified a variation in CACNA2D3 that disrupted a splice junction associated with ASD (A/G).
Wang et al .[47]	USA	(1)780 families with ASD (2)1204 affected subjects (3)6491 control subjects	CACNA1C	SNPs		genome-wide SNP genotyping	The result found an relationship between SNPs in CACNA1C and ASD
Cross-Disorder Group [9]	USA	(1)33,332ASD (2)27,888 controls	(1)CACNA1C (2)CACNB2	SNPs		genome-wide SNP genotyping	The result showed that SNPs within CACNA1C and CACNB2 were associated with ASD.
Hemara-Wahanui et al .[18]	New Zealand	27 families with ASD	CACNA1F	nucleotide substitution	gain-of-function	(1) DNA Sequencing (2) Functional Analyses	The molecular genetic analyses revealed an I745T CACNA1F allele in a New Zealand family, some male probands also affected with ASD.
Myers et al .[27]	USA	(1)142 ASD (2)143 SCZ (3) 240controls	CACNA1F	rare missense variant		DNA Sequencing	The study found a rare causal CACNA1F variants associated with ASD.
Palmieri et al .[32]	USA	(1)6ASD (2)6controls	Ca^2+^ concentration			Fluorimetric measure of Ca^2+^	The study found a significantly higher level of Ca^2+^ in ASD patients as compared to healthy controls.
Schmunk et al .[39]	USA	skin fibroblast cultures	IP3-mediated Ca ^2+^ release			High-throughput Ca^2+^ imaging	The study found a significantly reduction in IP3-mediated Ca2+ release from the ER.
Wen et al .[48]	USA	ASD genes				(1) Gene set enrichment analysis (2)Pathway-pathway interactions	The results showed that the process “calcium-PRC (protein kinase C)-Ras-Raf-MAPK/ERK” was a major contributor to ASD pathophysiology.
Skafidas et al .[40]	Australia	(1) Index sample:2609 ASD (2) Vlidation sample:737 ASD	CACNA1A	SNPs		Gene set enrichment analysis	The result showed that SNPs in CACNA1A to be among the top 15 SNPs contributing to the ASD diagnosis
Schmunk et al .[38]	USA	skin fibroblast cultures	IP3-mediated Ca ^2+^ release			High-throughput Ca^2+^ imaging	The study found a significantly depressed IP3-mediated Ca2+ signals in ASD.
O'Roak et al .[31]	USA	209 trios	(1)CACNA1D (2)CACNA1E	CNVs		Exome read-depth CNV analysis	The result showed that rare de novo alleles of CACNA1D and CACNA1E contributed to the genetic etiology of ASD.
De Rubeis et al .[11]	USA	(1) 3871 ASD (2)9937 controls	(1)CACNA2D3 (2) CACNA1D	de novo variant	loss of function	Exome sequencing	The result identified two de novo CACNA2D3 loss of function mutations in ASD cases and none in controls.
Girirajan et al .[17]	USA	(1) 2588 ASD (2) 580 controls	CACNA2D3	CNVs		Microarray analyses	The study reported an enrichment of CACNA2D3 deletion in ASD subjects compared to controls
Jiang et al. [22]	USA	32 families with ASD	CACNA1C	rare missense variant		Whole-genome sequencin	It found a rare missense mutation in CACNA1C (R1522Q) in a proband with ASD and an unaffected sibling.
Yuen et al. [51]	Canada	85 quartet families (parents and two ASD-affected siblings)	CACNB2	rare missense variant		Whole-genome sequencing	It found a CACNB2 (V2D) mutation in two affected siblings.
Yatsenko et al .[50]	USA	20 ASD	CACNA1B	duplication		(1) whole genome sequencing (2) custom 9q34 microarray	The study found a duplication of the chromosomal region 9q43.3, comprising the gene CACNA1B, in 12 out of 20 patients.
Prasad et al .[36]	USA	(1)696 unrelated ASD (2)1000 controls	CACNA2D4	CNVs		CGH microarray	The study identified multiple novel CNVs in ASD subjects, including the loss of CACNA2D4.

These included articles were published between 2004 and 2017, and nine of them were published in the last 5 years. Several early studies contained relatively small numbers of subjects, therefore, they might not have adequate statistical power to detect true risk alleles [26]. These included studies mainly originated in the USA (*n* = 19), followed by China, Germany, Australia and Canada (n = each 2) and New Zealand (n = 1). A series of genetic methods has been adopted to identify VGCC genetic variants associated with ASD, the most commonly used of which included next-generation sequencing, gene network analysis, and microarrays.

The search generated a total of 18 studies regarding the genetic association between Ca_v_ gene variants and ASD, 8 studies regarding the genetic association between auxiliary subunit gene variants and ASD, and 4 studies reported associations between calcium signaling pathways and ASD.

### Genetic associations between Ca_v_ genes and ASD

The search generated a total of 18 studies regarding the genetic association between Ca_v_ gene variants and ASD. Evidence generated from this review consistently implicated the role of Ca_v_ gene variants in the etiology of ASD.

The search yielded 3 studies that rendered CACNA1A a promising etiological candidate gene for ASD. Li et al. [23] examined the genetic relationship between CACNA1A and ASD in a Chinese Han population and reported an association between rs12609735 and rs7249246 in CACNA1A with ASD in a total of 553 trios, although this association would not survive after Bonferroni correction. Damaj et al. [10] identified 16 individuals carrying CACNA1A loss-of-function variants and presented ASD-like behavioral deficits by investigating four non-consanguineous families. The sequencing results revealed one CACNA1A gene deletion as well as two deleterious CACNA1A point mutations, including one known stop-gain and one new frameshift variant and a new splice site variant, which pointed to an association between CACNA1A variants and ASD symptoms. The study from Skafidas et al. [40] generated a predictive genetic classifier based on a linear function of 237 SNPs that distinguished ASD from controls and found that a SNP (rs10409541) in CACNA1A was among the top 15 most contributory SNPs for ASD diagnosis prediction.

The search returned only one study regarding the role of CACNA1B variants in the etiology of ASD. Yatsenko et al. [50] analyzed 20 patients with copy number gains involving the subtelomeric 9q34 region and reported a monogenic duplication of the CACNA1B gene in 12 out of 20 patients with a phenotype including ASD.

The search generated 7 studies that implicated CACNA1C as a susceptibility gene for ASD. The Cross-Disorder Group of the Psychiatric Genomics Consortium [9] compiled data on five major psychiatric disorders in a large genome-wide meta-analysis with 33,332 cases and 27,888 controls, rendering SNPs in CACNA1C a promising etiological candidate gene for five major psychiatric disorders, including ASD. Wang et al. [47] reported findings from a genetic analysis in a large number of ASD cases, which provided suggestive evidence of a relationship between SNPs in CACNA1C and ASD. The study of Splawski et al. [42] described the phenotypic characterization of Timothy syndrome (TS) and identified two analogous mutations, p.G406R and p.G402S in CACNA1C, which led to significantly impaired current inactivation of the Ca_v_1.2 splice form, raised the possibility that the ASD phenotypes associated with TS may result from the CACNA1C gain-of-function mutation. Smith et al. [41] conducted a detailed microarray analysis of 69 ASD probands and 35 parents and identified a rare homozygous deletion in a male proband that removed one copy of the CACNA1C calcium channel genes (12p13.33). Li et al. [23] examined the relationship between CACNA1C variants and ASD in 553 nuclear families of Chinese Han ancestry and found a nominal significant association between two SNPs (rs1006737 and rs4765905) in CACNA1C and ASD, suggesting that CACNA1C might play a role in the genetic etiology of ASD. A whole-genome sequencing study from Jiang et al. [22], who examined rare inherited genetic variants in 32 families with ASD, identified a rare missense mutation in CACNA1C in a proband with ASD and an unaffected sibling; therefore, it was unclear whether the mutation in CACNA1C was causative of ASD in this family. A genome-wide association study from Lu et al. [25] examined the potential role of VGCC variants in the etiology of ASD, rendering SNPs in CACNA1C a predisposing risk factor contributing to ASD.

Evidence from 4 studies proposed the role of CACNA1D variants in the genetic etiology of ASD. Pinggera et al. [33] functionally expressed two de novo mutations, p.A749G and p.G407R, in CACNA1D in tsA-201 cells to study their functional consequences using whole-cell patch-clamp analysis. The novel functional data strongly argued for an important role of CACNA1D gain-of-function mutations in the pathophysiology of ASD. Pinggera et al. [34] reported a de novo missense mutation in CACNA1D in a patient with ASD and examined the function of this mutation using whole-cell patch-clamp analysis, strengthening the evidence for CACNA1D gain-of-function mutations in the pathophysiology of ASD. O'Roak et al. [31] sequenced the exomes of 209 parent-child trios and identified rare de novo alleles of CACNA1D as top de novo risk mutations for ASD. De Rubeis et al. [11] conducted the largest ASD WES study to date to identify rare coding variants in 3871 ASD subjects and 9937 controls and identified five mutations located in CACNA1D, including G407R and A749G, in ASD subjects.

The search yielded only one study regarding the role of CACNA1E variants in the etiology of ASD. The study by O'Roak et al. [31] sequenced the exomes of 209 parent-child trios and identified de novo mutations in CACNA1E that contributed to the genetic etiology of ASD.

Two studies reported the potential role of CACNA1F variants in the etiology of ASD. Myers et al. [27] presented results from a large-sample resequencing study of candidate genes coupled with population genetics to identify rare variants associated with ASD and found a significant excess of rare missense CACNA1F variants in the cohort of ASD patients. The study of Hemara-Wahanui et al. [18] identified a CACNA1F mutation in a family with inherited night blindness, with some of the male members being affected with ASD. The mutation significantly affected the gating properties of the Ca_v_ 1.4 channel when exogenously expressed in tsA-201 cells, indicating that the molecular mechanism of the pathology was likely to involve a gain, rather than loss, of Ca_v_ 1.4 channel function.

The search yielded 2 studies examining the association between CACNA1G variants and ASD. Strom et al. [45] examined the association between variants in the chromosomal interval 17q11-q21 and ASD in 284 independent trios and identified the calcium channel subunit gene CACNA1G as a novel candidate gene for ASD. Lu et al. [25] examined the potential role of calcium channel genes in ASD by focusing on 10 genes that encode α 1 subunits in a cohort of 2781 parent/affected child trios and identified SNPs in CACNA1G as predisposing risk factors contributing to ASD.

The search returned one study regarding the role of CACNA1H variants in the genetic etiology of ASD. Splawski et al. [43] examined the role of calcium channel gene mutations in the pathogenesis of ASD and identified missense mutations in CACNA1H in 6 of 461 subjects with ASD that resulted in decreased activity of the Ca_v_3.2 channel, implicating the role of CACNA1H loss-of-function mutations in the development of ASD. However, some of the mutations were also present in unaffected family members, indicating that the mutations were not fully penetrant.

The search yielded only one study regarding the role of CACNA1I variants in the development of ASD. Lu et al. [25] examined the role of VGCC variants in the development of ASD by focusing on 10 genes that encode α1 subunits and identified SNPs in CACNA1I as genetic risks for ASD.

### Genetic associations between auxiliary subunit genes and ASD

Given that the auxiliary subunits Ca_v_α2δ and Ca_v_β have profound effects on the biophysical properties and membrane targeting of the Ca_v_α1 subunit, genes encoding these subunits may also be linked to ASD. The search generated a total of 8 studies regarding the genetic association between auxiliary subunit gene variants and ASD. Existing evidence suggests that variants in the gene encode the auxiliary subunits Ca_v_α2δ and Ca_v_β, which also contribute to the pathogenesis of ASD.

The search yielded 3 studies that reported an association between CACNB2 variants and ASD. Breitenkamp et al. [5] sequenced the exons and flanking introns of CACNB2 in 155 ASD subjects and 259 unaffected controls and identified three missense variants in the coding region of CACNB2 (G167S, S197F, and F240L) in ASD probands that resulted in decelerated inactivation of the Ca_v_1.2 subunit, supporting the role of CACNB2 gain-of-function mutations in the pathophysiology of ASD. In line with these results, a study from the Psychiatric Genomics Consortium (2013), which analyzed genome-wide single-nucleotide polymorphism (SNP) data for the five disorders, identified CACNB2 as a risk locus for these five major psychiatric disorders, including ASD. An exome sequencing study by Yuen et al. [51] investigating 85 quartet families in which two siblings were affected with ASD to identify ASD-related gene variants found a CACNB2 (V2D) mutation in two affected siblings.

The search generated 3 studies regarding the role of CACNA2D3 variants in the development of ASD. Iossifov et al. [21] reported on the sequence analysis of whole exomes from 343 families, each with a single ASD child and at least one unaffected sibling. The results from exome sequencing showed an excess of de novo splice site mutations in CACNA2D3, one of many identified gene-disrupting mutations, in ASD subjects compared to unaffected siblings. De Rubeis et al. [11] identified rare coding variants in 3871 ASD subjects and 9937 ancestry-matched or parental controls and implicated CACNA2D3 as a risk gene following the identification of two de novo loss-of-function mutations in cases and none in controls. A copy number variation (CNV) study from Girirajan et al. [17], who comprehensively characterized recurrent CNVs for both large and putative smaller hotspots in 2588 ASD subjects and 580 controls, reported an enrichment of CACNA2D3 deletion in ASD subjects compared to controls.

The search returned 2 studies regarding the association between CACNA2D4 variants and ASD. Smith et al. [41] conducted a detailed microarray analysis of 69 ASD probands and 35 parents and identified a rare homozygous deletion in a male proband that removed one copy of the CACNA2D4 calcium channel genes (12p13.33). Prasad et al. [36] examined de novo copy number variations (CNVs) through combined analysis of CGH and SNP array data sets in a cohort of 696 unrelated ASD subjects and 1000 controls. The high-resolution CGH data identified multiple novel CNVs in ASD subjects, including the loss of CACNA2D4.

### Associations between calcium signaling pathways and ASD

The genetic contributions of VGCC variants to the pathogenesis of ASD may arise from functional disturbances in a variety of signaling cascades in which these genes are involved, since it is the combination of these factors that ultimately shapes the phenotype. Calcium signaling pathways may serve as scaffolds that unite genetic lesions into a consensus etiology of ASD. Identification of variants in calcium channel genes led to the discovery of molecular signaling pathways involved in ASD, including the IP3-Ca^2+^ pathway and the MAPK pathway, providing further insights into the etiology of ASD.

The inositol triphosphate receptor (IP3R) forms a calcium-permeable ion channel located in the membrane of the endoplasmic reticulum (ER) [29]. The IP3 pathway changes intracellular Ca^2+^ concentration by releasing calcium stored in the ER, which is critical for synaptic plasticity, neuronal excitability, neurotransmitter release, and axonal growth, demonstrating the central integration position of IP3R in neurons [19, 46]. Suppressed IP3-mediated calcium signaling has been linked to ASD etiology in two studies. Schmunk et al. [38] evaluated functional deficits in IP3-mediated Ca^2+^ signaling from three monogenic models of ASD and identified significantly depressed IP3-mediated Ca^2+^ signals in ASD. Schmunk et al. [39] further extended these findings to a cohort of sporadic ASD patients and reported a significant reduction in IP3-mediated Ca^2+^ release from the ER. Recent research has implicated the abnormal MAPK pathway as a possible molecular mechanism of ASD. Wen et al. [48] performed a systematic analysis of the ASD pathway network and reported a high level of involvement of the MAPK signaling pathway together with calcium channel genes in the ASD pathway network. One study proposed a role of abnormal calcium homeostasis in the etiology of ASD. Palmieri et al. [32] examined the Ca^2+^ concentrations in the postmortem temporocortical gray matter of six matched patient-control pairs and reported a significantly higher level of Ca^2+^ in ASD patients than in healthy controls, supporting the involvement of altered Ca^2+^ homeostasis in the etiological pathway of ASD.

## Discussion

The identification of VGCC genetic variants as risk factors for ASD may not only be extracted from the ability of calcium channels to mediate Ca^2+^ into neurons, which can trigger many calcium-modulated functions, but also derive from the role of calcium channels as signaling hubs, which can link together different cellular signaling pathways.

The present review summarized recent works regarding the genetic association between VGCCs and ASD. The most prominent VGCC variants found in ASD were those falling within loci encoding the α subunits CACNA1A, CACNA1B, CACNA1C, CACNA1D, CACNA1E, CACNA1F, CACNA1G, CACNA1H, and CACNA1I as well as those of their accessory subunits CACNB2, CACNA2D3, and CACNA2D4. Two signaling pathways, the IP3-Ca^2+^ pathway and the MAPK pathway, were identified as scaffolds that united genetic lesions into a consensus etiology of ASD.

Structure-function analyses involving the introduction of gene variants into cloned VGCCs have provided important clues concerning gain-of-function and loss-of-function properties of these calcium channels. Variants located in CACNA1C, CACNA1D, CACNA1F and CACNB2 cause gain of function by preventing voltage-dependent inactivation of Ca_v_1.2, Ca_v_1.3, Ca_v_1.4, and Ca_v_β2, leading to excessive influx of Ca^2+^. Variants located in CACNA1A and CACNA1H turned out to rather cause loss of function in Ca_v_2.1 and Ca_v_3.2 due to reduced conductance and shifted voltage dependence of activation, resulting in decreased channel activity. It is difficult to form a clear consensus concerning gain or loss of function of the VGCC variants associated with ASD, which forms the basis of this review. It is conceivable that dysregulation of these calcium channels in either direction could result in functional and developmental abnormalities considering the requirement for precise regulation of internal Ca^2+^ concentrations for normal cell signaling and gene transcription during development.

Cytosolic calcium signals originate from either the influx of extracellular Ca^2+^ or the release of intracellular Ca^2+^ stored in the ER. The mitochondria actively communicate with the ER calcium signaling apparatus in the generation of rapid calcium signals, forming a bidirectional link between energy metabolism and transmitted cellular signals. The interaction of ER-mitochondria in the control of calcium homeostasis implies that calcium signaling may serve as a scaffold that integrates VGCC genetic lesions and organelle dysfunction, including the ER and mitochondria, into a consensus pathophysiology of ASD.

Given the consistent evidence generated from this review supporting the role of VGCC genetic variants in the pathophysiology of ASD, it is worthwhile to consider targeting Ca_v_α1, Ca_v_α2δ, and Ca_v_β subunits as a potential therapeutic strategy to treat this disorder. Despite the existence of several drugs targeting Ca_v_α1 and Ca_v_α2δ subunits, they are mainly used in the treatment of stroke, epilepsy, and cardiovascular disease [52]. Lamotrigine, a drug that blocks Ca_v_2.3 channels, has been used to treat bipolar disorder (BD) [37]. Z160, a drug that blocks the Ca_v_2.2 channel, has shown some promise in treating anxiety [53]. Calcium channel blockers are promising for the treatment of ASD, but gain-of-function mutations are in many cases less sensitive to blocking because of the loss of inactivation [20, 42].

## Conclusion

Findings generated from this review proposed that the genetic component of ASD may involve a combination of multiple common alleles in the VGCC gene, represented by SNPs, each with a relatively small impact, together with a few rare alleles in the VGCC gene, represented by CNVs and deleterious mutations, which might produce a relatively large increased risk. The phenotype of ASD may be conferred by the sum of genetic risks with regard to both rare and common alleles in the VGCC gene together with risks from environmental stimuli.

Given the consistent evidence generated from this review supporting A total of 1163 records were generated from the initial search of these three databases. After eliminating 269 duplicates, 894 articles considered as potentially relevant for subsequent analysis. The titles and abstracts of all non-duplicated papers were preliminary checked by two authors, 803 articles were excluded as lacking of relevance. The full-text of the leaving 91 citations were further appraised independently for eligibility by two authors, 63 articles were excluded as failing to meet the selection criteria. A final total of 28 articles were selected as source documents for this review. Genetic variants in the pathophysiology of ASD, it is of great value to examine whether existing FDA-approved drugs modulate Ca^2+^ signaling function in the treatment of ASD. Although these findings have confirmed the genetic associations between VGCCs and ASD, the underlying mechanisms by which these variants produce the ASD phenotypes have not been characterized. Existing evidence implicated Ca^2+^ signaling as the most relevant node of an integrative network model for gene-environment interactions in the ASD context [54]. Further studies should shift attention from the role of individual variants to the compounded impacts of different variants as they interact with other genes and the environment.

## Data Availability

Not applicable.

## Abbreviations

ASD: Autism Spectrum Disorder
VGCCs: Voltage-Gated Calcium Channels
HVA: High voltage-activated channels (HVA)
LVA: Low voltage-activated channels
SNPs: Single nucleotide polymorphisms
IP3R: Inositol triphosphate receptor
ER: Endoplasmic reticulum
VDI: Voltage- dependent inactivation
CDI: Calcium-dependent inactivation
BD: Bipolar Disorder
CNVs: Copy number variations
